# Comprehensive Analysis of Gastrointestinal Injury Induced by Nonsteroidal Anti-Inflammatory Drugs Using Data from FDA Adverse Event Reporting System Database

**DOI:** 10.3390/ph18081204

**Published:** 2025-08-14

**Authors:** Motoki Kei, Yoshihiro Uesawa

**Affiliations:** 1 Department of Medical Molecular Informatics, Meiji Pharmaceutical University, Tokyo 204-8588, Japan; 2 Department of Pharmacy, Tokyo Women’s Medical University Adachi Medical Center, 4-33-1 Kohoku, Adachi-ku, Tokyo 123-8558, Japan

**Keywords:** NSAIDs, gastrointestinal disorders, FAERS database, principal component analysis, hierarchical cluster analysis

## Abstract

**Background/Objectives:** Nonsteroidal anti-inflammatory drugs (NSAIDs) are commonly associated with gastrointestinal (GI) adverse events. This study aimed to assess the incidence and patterns of NSAID-induced GI disorders using the FDA Adverse Event Reporting System (FAERS) database and to compare the risks among different NSAIDs. **Methods:** NSAID-related reports were extracted from FAERS, focusing on 21 ulcer-related GI events with ≥1000 reports each, based on MedDRA v26.0. The number of reports, reporting odds ratios, and *p*-values were calculated and visualized using a volcano plot. Principal component analysis(PCA) was carried out to reduce the dimensionality of the dataset and revealed under-lying patterns in the data.PCA was performed to identify patterns related to risk, severity, and injury site, whereas hierarchical clustering was used to group NSAIDs based on these patterns. Hierarchical cluster analysis is a method of grouping similar data to generate a classification. **Results:** Statistically significant signals were identified for 19 of the 21 GI-related adverse events, including the serious condition of perforation. PCA revealed that the first component represented risk, the second severity, and the third the site of injury (upper vs. lower GI tract). Cyclooxygenase-2 (COX-2) selective NSAIDs (e.g., celecoxib, rofecoxib) were associated with a lower incidence but greater severity, primarily in the upper GI tract. Conversely, nonselective NSAIDs (e.g., acetylsalicylic acid, lornoxicam) showed higher incidence rates, though the events were generally milder. In our dataset, acetylsalicylic acid had the highest incidence, whereas meloxicam showed the highest severity. Clustering analysis revealed three distinct NSAID groups with differing patterns in risk, severity, and affected GI site. Mild adverse events may be underreported in FAERS. Dosage-related effects were not assessed in this study. **Conclusions:** NSAIDs differ significantly in their gastrointestinal adverse event profiles, attributable to COX selectivity. When selecting an NSAID, both the likelihood and the nature of potential GI harm should be considered.

## 1. Introduction

Nonsteroidal anti-inflammatory drugs (NSAIDs) are widely used to reduce pain and inflammation, and several types of NSAIDs are included in the list of essential medicines of the World Health Organization [1]. However, NSAIDs cause a broad range of adverse events that affect various organ systems, most commonly the renal, gastrointestinal (GI), and cardiovascular systems [2]. The primary mechanism of several NSAID-induced adverse effects involves cyclooxygenase (COX) inhibition, which reduces protective prostaglandins and can lead to oxidative stress [2]. In recent years, there have been increasing concerns regarding the cardiovascular and GI risks associated with NSAIDs. In October 2024, the Japanese Ministry of Health, Labour and Welfare issued a safety notice revising the precautions for systemic NSAIDs after a national database study showed that these medications were associated with an increased risk of myocardial infarction and stroke [3]. NSAIDs account for approximately 30% of adverse event-related hospitalizations, and their use has been associated with serious cardiovascular, GI, and renal complications [3]. Further, according to 1997 statistics, NSAID-related upper-gastrointestinal complications cause 5000–16,500 deaths per year in the United States [4,5]. In the United Kingdom, a 1999 report indicates that, on average, 1 in 1200 patients taking NSAIDs for at least two months will die from NSAID-related upper-gastrointestinal complications who would not have died otherwise, extrapolating to about 2000 deaths each year [6]. These data underscore the importance of preventing and managing GI injury induced by NSAIDs. The main mechanism of NSAID-related GI injury is the inhibition of COX-1, which suppresses prostaglandin-mediated mucosal protection and predisposes the gastric lining to damage [7]. Helicobacter pylori infection further worsens this risk, and it has a synergistic effect with NSAIDs, thereby significantly increasing the risk of peptic ulcer bleeding in NSAID users [7]. A previous meta-analysis reported that the odds of peptic ulcer disease are approximately 61-fold higher in patients using NSAIDs and those with H. pylori infection than in individuals with neither risk factor. Meanwhile, the odds are about 19-fold higher only in those taking NSAIDs [8]. Several studies have quantified the differences in the risk of GI disorders among various NSAIDs. For example, based on a previous analysis, the incidence of GI bleeding ranged from approximately 0.4% to 1.7% in conventional NSAIDs and from approximately 0.3% to 0.8% in COX-2-selective NSAIDs [9]. These adverse events are widely recognized, and prior studies have examined the cardiovascular and renal risks of specific NSAIDs such as celecoxib, naproxen, and ibuprofen [10]. In the APPROVe trial, use of the COX-2-selective inhibitor rofecoxib was associated with roughly a two-fold increase in major cardiovascular events versus a placebo, leading to its withdrawal from markets worldwide on 30 September 2004 [11]. Several meta-analyses have compared the risk of major GI complications across individual NSAIDs. A combined analysis of 12 studies found that ibuprofen had the lowest relative risk of major GI events among NSAID users, followed in ascending order by diclofenac, acetylsalicylic acid, naproxen, indomethacin, piroxicam, and ketoprofen [12]. The risk differences were significantly attributed to the dosages used in clinical practice (with ibuprofen considered low-risk, in part, because it is often used at lower doses) [12]. In general, several studies have consistently reported that ibuprofen and diclofenac are associated with a lower risk of GI disorders. Meanwhile, ketoprofen, piroxicam, and azapropazone are associated with a higher risk. COX-2-selective NSAIDs (e.g., celecoxib and rofecoxib) cause fewer GI injuries than nonselective NSAIDs, particularly in high-risk patients, thereby resulting in a significantly reduced overall GI injury [13]. A clear dose-dependence has also been observed, with higher NSAID doses increasing the risk of GI complications by approximately 2- to 3-fold compared with lower doses [13]. Despite these clinical insights, a comprehensive data-driven comparison of GI risk across the spectrum of NSAIDs has not been conducted. Currently, there is no clear evidence-based guidance for selecting various NSAIDs with respect to GI safety. In light of this, the current study comprehensively evaluated the inter-drug differences and patterns in the risk of NSAID-induced GI disorders using data from the FDA Adverse Event Reporting System (FAERS) database.

## 2. Results

### 2.1. Generation of Table Data

**Figure 1:**
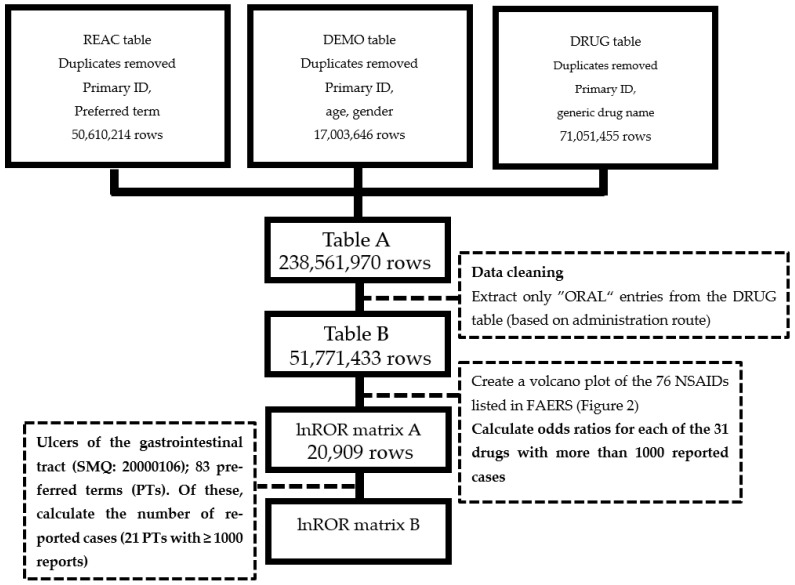
Flowchart for creating tables for the analysis.

After data preprocessing, the final analysis dataset (Table B in Figure 1) was constructed, as described in Section 4. We then calculated the total number of reported GI ulcer cases for each NSAID that was included in this analysis. Table 1 presents the results. In addition, for each NSAID–event pair, the reporting odds ratio (ROR) and its *p*-value were calculated. The log-transformed ROR values (lnROR matrix B) were used as the input for the principal component analysis (PCA) (Figure 1).

### 2.2. Association of Age and Sex with GI Ulcers

As shown in Table 2, female patients had a slightly higher reporting risk of peptic ulcer than male patients. Although this difference was statistically significant, the estimated odds were nearly similar between sexes; given the potential FAERS reporting bias, we interpreted this as not a clinically meaningful sex difference. In contrast, older patients (aged ≥70 years) exhibited a significantly higher risk of GI ulcer events than younger ones, potentially reflecting age-related declines in mucosal defense.

### 2.3. Volcano Plot

**Figure 2:**
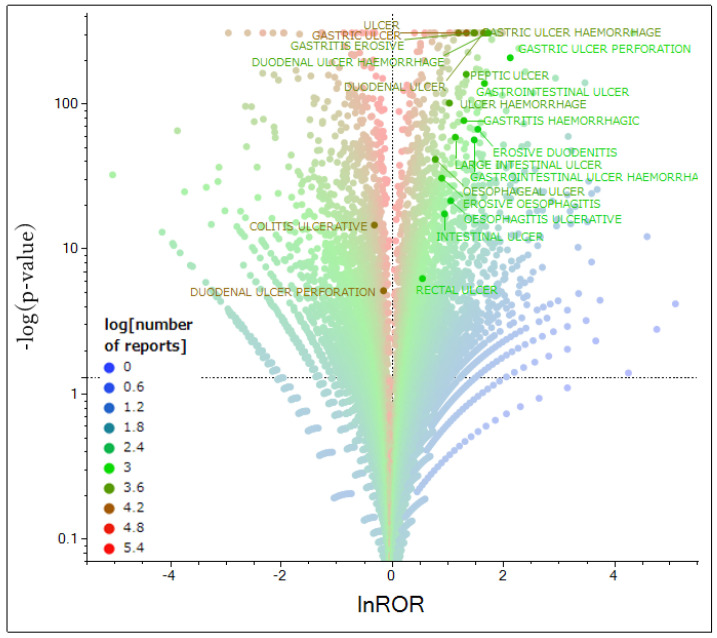
Volcano plot for NSAIDs. The vertical axis represents statistical significance based on Fisher’s exact test, whereas the horizontal axis represents the risk of inducing adverse reactions. Drugs significantly associated with adverse reactions appear in the upper right corner. The scatterplot is color-coded for antineoplastic agents according to the number of drug reports. Adverse reactions with lnROR ≥ 1 and −log *p* ≥ 1.3 are labeled with the corresponding drug name.

A volcano plot was created to visualize NSAID-disproportionality signals for GI adverse events (Figure 2). In total, 19 of the 21 analyzed GI ulcer event types had statistically significant signals.

### 2.4. Principal Component Analysis

**Figure 3:**
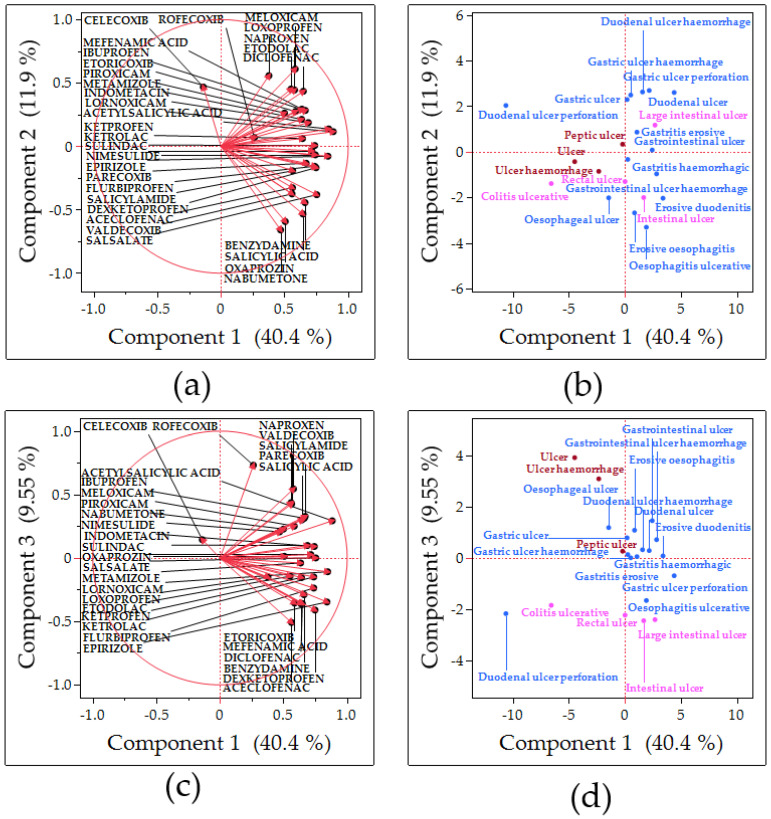
Association of gastrointestinal ulcers with NSAIDs based on the PCA. Loading plots (**a**,**c**) present the association between NSAIDs and each principal component. Each loading vector represents NSAIDs. Score plots (**b**,**d**) show the association between adverse events related to gastrointestinal ulcers and each principal component. Each dot represents an adverse effect. Each plot is colored by lnROR based on the calculations for all drugs.

**Figure 4:**
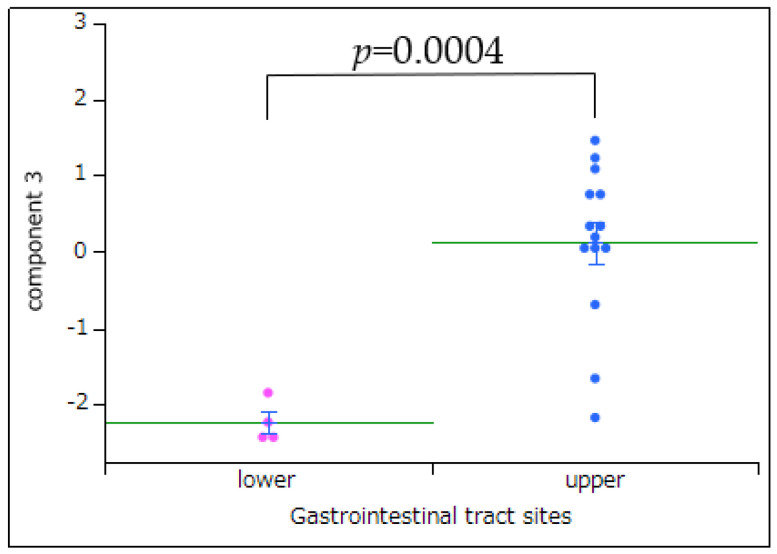
Bivariate association between PC3 and sites of GI tract injury. This figure illustrates the relationship between PC3, derived from PCA, and the anatomical site of GI injury. The green line indicates the average PC3 score for each category (upper vs. lower GI tract), whereas the blue error bars represent the standard deviation, conveying the variability around the mean. A significant difference in PC3 scores was observed between upper and lower-GI events (*p* = 0.0002), supporting the interpretation that PC3 reflects the site of injury. Positive PC3 values are associated with upper-GI injuries, while negative values are associated with lower-GI injuries.

The PCA identified three major components, with the first, second, and third components accounting for 40.4%, 11.9%, and 9.5% of the variance, respectively (Figure 3). The first principal component (PC1) was interpreted as representing the overall risk of adverse event occurrence. The third principal component (PC3) distinguished upper from lower-GI events: the PC3 scores differed significantly according to the location of adverse events (upper vs. lower GI, *p* = 0.0002, *t*-test; Figure 4). Table S1 depicts the detailed component loadings for each drug.

### 2.5. Hierarchical Cluster Analysis

**Figure 5:**
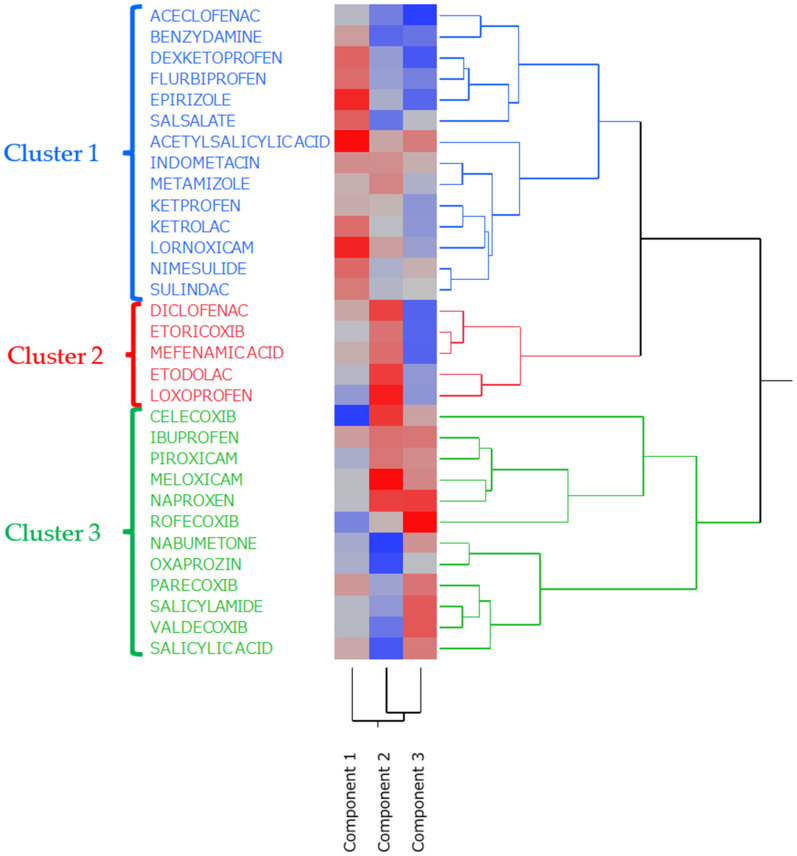
HCA. The figure presents the association between the three principal components and the 31 NSAIDs. In the color map, the red color indicates positive correlations, and the blue color represents negative correlations.

As shown in Figure 5, via hierarchical cluster analysis (HCA), the 31 NSAIDs were grouped into three clusters based on their PCA loadings. For example, cluster 1 comprised drugs such as acetylsalicylic acid, indometacin, and lornoxicam. These NSAIDs are nonselective agents characterized by a high estimated risk of GI events (high PC1 scores), a reduced severity of adverse events (low PC2 scores), and a tendency to cause lower-GI tract injury (negative PC3 scores). In contrast, cluster 3 primarily included COX-2-selective NSAIDs such as celecoxib, rofecoxib, parecoxib, and valdecoxib. Drugs in this cluster were characterized by a lower overall risk of adverse events (low PC1) and a greater severity of adverse events (high PC2). Further, they were more likely to affect the upper GI tract (positive PC3). Cluster 2 comprised the remaining NSAIDs (e.g., diclofenac, etoricoxib) and exhibited intermediate characteristics: a generally low risk of adverse events, similar to cluster 3, a higher severity of adverse events, similar to cluster 3, and a tendency toward lower-GI injury, similar to cluster 1. In summary, the clustering analysis differentiated groups of NSAIDs with distinct profiles in terms of the risk, severity, and affected site of GI events.

## 3. Discussion

### 3.1. Association of Age and Sex with GI Ulcers

As shown in Table 2, older patients had a significantly higher risk of NSAID-associated GI disorders, possibly due to age-related declines in mucosal defense mechanisms. There was no significant difference in terms of risk according to sex. Therefore, a similar level of caution is required for both male and female patients.

### 3.2. Volcano Plot

The volcano plot analysis showed that 19 of the 21 GI ulcer event types had significant signals associated with the use of NSAIDs. Notably, signals in the upper-right quadrant (indicating a high ROR and a high significance) corresponded to severe upper-GI events, such as gastric ulcer perforation, duodenal ulcer, hemorrhagic gastric ulcer, erosive gastritis, and hemorrhagic duodenal ulcer. Based on this finding, NSAIDs with a high propensity to cause GI adverse events were more likely to be associated with more severe manifestations (deep ulcers, bleeding, and perforation). Recognizing these drug-specific risk patterns may help avoid the use of NSAIDs that trigger the most severe adverse events. Of the analyzed ulcer terms, only two (ulcerative colitis and perforated duodenal ulcer) had no clear signal. Ulcerative colitis is an autoimmune chronic inflammatory disease not directly caused by NSAIDs. Thus, this notion likely explains the lack of signals. In the case of the perforated duodenal ulcer, the statistical power for detecting a signal was low relative to other GI events. Thus, reporting biases (as discussed in Section 3.5) might have obscured an association.

### 3.3. PCA

PCA was carried out to reduce the dimensionality of the dataset and revealed underlying patterns in the data [14].

#### 3.3.1. PC1

PC1 was interpreted as an index of overall GI risk. In the PCA loading plots, all NSAIDs, except celecoxib, had positive PC1 loadings. Hence, higher PC1 values correspond to a greater risk of GI adverse events. Acetylsalicylic acid, lornoxicam, and epirizole had the highest PC1 scores. Meanwhile, celecoxib and rofecoxib, which are both COX-2 selective, had the lowest PC1 scores. This pattern indicates that the risk captured by PC1 is linked to the inhibitory activity of COX-1. In particular, drugs with a stronger COX-1 inhibition were more likely to have a higher GI risk. Consistently, 15 of the 21 GI ulcer terms had positive scores on PC1 in the score plot for adverse events. Adverse events with high PC1 scores included perforated gastric ulcer and erosive duodenitis. Hence, PC1 is associated with ulcerative outcomes likely caused by COX-1 inhibition.

#### 3.3.2. Second Principal Component (PC2)

From the score plot, the positive direction of PC2 indicates lesions with a high risk of deep ulcers and acute complications (bleeding and perforation). Meanwhile, the negative direction indicates superficial lesions such as erosive esophagitis and intestinal lesions. NSAIDs inhibit mucosal prostaglandin synthesis via COX-1 inhibition, which reduces GI mucosal defenses. As a result, gastric and duodenal ulcers may progress, leading to vascular erosion and perforation. In fact, the incidence of major complications (such as bleeding and perforation) of peptic ulcers is approximately five times higher in NSAID users than in nonusers [7]. The prevalence of peptic ulcers in patients using NSAIDs is 15–40%, and the risk of gastric ulcers is slightly higher than that of duodenal ulcers [4]. By contrast, esophageal ulcers caused by NSAIDs alone are rare. In fact, esophageal ulcers that are caused by NSAIDs have only been reported sporadically on a case report basis [15]. In terms of frequency, NSAID-related esophageal ulcers are significantly less common than NSAID-related gastric and duodenal ulcers. From a pathological point of view, gastric and duodenal ulcers are caused by both aggressive factors, such as gastric acid, and defensive factors, such as prostaglandins. However, the pathology of esophageal ulcers is somewhat different. NSAIDs decrease the lower esophageal sphincter tone and esophageal peristalsis, causing reflux of gastric contents. This can lead to chronic exposure of the esophageal mucosa to gastric acid, resulting in Barrett’s esophagus and esophageal stricture [16]. In the positive direction of PC2, bleeding and perforated ulcers are included, thereby indicating more severe adverse events. By contrast, the negative direction includes esophagitis and intestinal lesions, which are predominantly mucosal injuries and present a relatively low risk for acute complications. If a person using NSAIDs takes tablets without adequate fluids, or they take the drugs before bedtime, the tablets may come into prolonged contact with the esophageal mucosa, causing local mucosal injury and localized ulcers [17,18,19]. This phenomenon is referred to as drug-induced esophagitis (pill esophagitis). NSAIDs, bisphosphonates, and some antibiotics are known as causative agents [18]. In a previous report on ibuprofen-induced esophageal ulceration, the characteristic endoscopic findings of NSAID-induced ulceration were large, shallow, solitary ulcers in the central esophagus near the aortic arch with a normal surrounding mucosa. These findings indicated that mucosal contact with the NSAIDs caused the disorder [15,20]. Thus, esophageal mucosal damage attributed to direct stimulation by NSAIDs themselves is a possible mechanism [20]. In addition, NSAIDs may indirectly exacerbate gastroesophageal reflux disease (GERD) by suppressing the production of prostaglandins, leading to gastric acid overproduction and the stagnation of gastric contents [16], and the risk of GERD symptoms such as heartburn and acid reflux is approximately twice as high in patients taking NSAIDs than in those not taking them. Moreover, patients may present with reflux esophagitis [19]. NSAIDs themselves are not a decisive factor in inducing new-onset GERD in healthy individuals. However, they can intensify it if mild reflux is already present [19]. Although NSAIDs themselves are not a decisive factor in inducing new-onset GERD in healthy individuals, they can intensify it in patients with mild reflux [19]. NSAIDs aggravate esophageal sphincter tone and peristalsis in some cases, and the relaxation and decreased motility of the sphincter muscles may facilitate reflux of gastric contents into the esophagus [16]. Thus, NSAIDs may indirectly cause esophageal mucosal injury (erosions and ulcers) not only via direct mucosal irritation, but also via the aggravation of GERD. NSAIDs decrease the production of mucoprotective prostaglandins by inhibiting COX-1 and the mucus barrier in the stomach and small intestine. The chronic use of NSAIDs causes small intestinal stenosis and Crohn’s disease-like narrowing. Erosions, ulcers, and hemorrhage of the small intestine have been frequently observed, thereby indicating increased permeability. NSAIDs can cause enteropathy and colonic disease via systemic effects on the mucosa. COX inhibition in the gut increases mucosal permeability and decreases mucosal blood flow, thereby allowing gut bacteria and toxins to induce inflammation. This phenomenon results in NSAID-induced colitis, small-intestine narrowing, and small-intestine ulceration. These lesions often present with nonspecific symptoms (such as abdominal pain, diarrhea, and anemia) [7]. Colitis caused by NSAIDs can lead to chronic bleeding and may be associated with iron deficiency anemia. However, significant bleeding and perforation, as observed in peptic ulcers, are considered rare [7,17]. NSAIDs inhibit COX-1, which reduces gastric mucosal defense and promotes ulcer formation when combined with gastric acid. These ulcers can penetrate deep into the gastric mucosa and cause ulcer formation. These ulcers are likely to reach substantial depths and cause bleeding and perforation if left untreated. Moreover, these lesions are relatively superficial, and the risk of acute perforation or massive bleeding is low [7]. Based on the abovementioned data, PC2 refers to NSAID-related injuries located in the esophagus and colon, which are more likely to be superficial, location-specific (often by local contact or systemic bowel action) and less likely to cause acute fatal bleeding/perforation. The positive direction of PC2 was inferred to be acid-dependent prostaglandin-losing ulcers. Meanwhile, the negative direction was inferred to be direct irritant or permeability-mediated lesion throughout the digestive tract.

Based on the abovementioned data, PC2 is a component from which information regarding the severity of GI tract injury was extracted. According to the loading plot, meloxicam, loxoprofen, and celecoxib had the highest estimated severity. By contrast, nabumetone and oxaprozin had the lowest estimated severity.

#### 3.3.3. PC3

PC3 corresponded to the anatomical site of injury (upper vs. lower GI tract). Consistent with this finding, PC3 scores differed significantly between the upper and lower-GI events (*t*-test, *p* = 0.0002). Thus, PC3 was interpreted as the component reflecting the site of NSAID-induced damage. Based on the loading plot, the NSAIDs with high positive PC3 loadings (more associated with upper-GI injury) included rofecoxib, naproxen, and valdecoxib. Meanwhile, the NSAIDs with high negative loadings (more associated with lower-GI injury) included aceclofenac, dexketoprofen, and mefenamic acid. Similarly, according to the score plot for adverse events, terms such as “ulcer” and “ulcer hemorrhage” ranked highest on PC3, thereby indicating the involvement of upper-GI lesions. COX-1 and COX-2 enzymes are distributed throughout the GI tract (with a particularly high COX-2 expression in the ileum of the lower GI) [21]. NSAID-induced GI injury is primarily driven by COX-1 inhibition. Thus, the pattern captured by PC3 likely reflects how an NSAID’s COX selectivity influences the injury site. Therefore, PC3 distinguishes NSAIDs based on whether their GI toxicity manifests more in the upper or lower GI tract, which is determined by the drug’s COX-1/COX-2 inhibitory profile.

### 3.4. Hierarchical Cluster Analysis

The clustering results can be understood with consideration of the PCA findings. Cluster 1 (e.g., acetylsalicylic acid, indometacin, and lornoxicam) comprised COX-nonselective NSAIDs characterized by a high risk of adverse event occurrence (high PC1 scores), a reduced severity of adverse events (low PC2 scores), and predominantly lower GI tract effects (negative PC3 scores). Cluster 2 (e.g., diclofenac, etoricoxib) included a mix of nonselective and COX-2-selective NSAIDs characterized by a lower risk of adverse event occurrence (low PC1) and a relatively higher severity (moderately high PC2). Similarly to cluster 1, these showed a tendency toward lower GI involvement (negative PC3). Cluster 3 (e.g., celecoxib, rofecoxib, parecoxib, and valdecoxib) was primarily composed of COX-2-selective NSAIDs characterized by a low risk of occurrence (low PC1), a greater severity of adverse events (high PC2), and predominantly upper-GI tract effects (positive PC3). In summary, each cluster of NSAIDs exhibited a distinct profile in terms of risk, severity, and affected site, indicating that the drugs were successfully categorized into clinically significant risk groups.

### 3.5. Limitations

This study had several limitations. First, FAERS is a spontaneous reporting database of adverse drug reactions. Spontaneous reporting of adverse drug reactions is subject to reporting biases, including underreporting and missing or incorrect data. In addition, because the denominator of drug users is unknown, the actual incidence rate cannot be calculated, and an absolute risk assessment cannot be performed. As a countermeasure, the number of drugs detected was limited by the number of reports. In addition to underreporting, other biases have also been observed. The notoriety effect refers to an increase in the number of reports on newly identified adverse events. The spillover effect refers to an increase in the number of reports on the same type and indication of a particular drug that became the focus of adverse event monitoring. The Weber effects refer to patterns in which the number of reports increases immediately after marketing and then decreases over time. These biases may be associated with reporting under- or overestimation. Another bias is the masking effect, in which certain adverse events are underestimated because of their association with other drugs [22]. Another concern is the difficulty in identifying the drug responsible for an adverse reaction when multiple medications have been administered [23,24]. Further, although not included in this analysis, information such as the underlying disease, concomitant medications, number of medications, method of administration, and duration of administration may be confounding factors that influence the development of adverse effects. Nevertheless, we believe that future studies can provide insights that consider these factors.

## 4. Materials and Methods

### 4.1. Database

Data from the FAERS database were used to conduct a comprehensive analysis of NSAID-associated GI disorders. We used the FAERS database, which contains cases from January 2004 to March 2023 (May 2023 public version). FAERS is a large pharmacovigilance database maintained by the Food and Drug Administration [25]. It comprises seven linked tables: DEMO (demographic characteristics of the patients such as age, sex, and weight), DRUG (drug information, including name and administration route), REAC (reported adverse events by preferred term), OUTC (outcomes), RPSR (report sources), INDI (indication), and THER (therapy details). Drug names were standardized to their generic forms (curation provided by INTAGE Healthcare Inc., Tokyo, Japan). To prepare our dataset, the DRUG, REAC, and DEMO tables were merged using their primary IDs, and duplicate records were removed to create Table A. From Table A, we only extracted cases with an oral route of administration to create a subset (Table B) for analysis. The requirement for ethical approval and informed consent was waived by the Ethics Committee of Meiji Pharmaceutical University because this study used anonymized data from an open-access database.

### 4.2. Adverse Events and Analysis of the Participants

The NSAIDs included in this study were defined according to the Anatomical Therapeutic Chemical classification system of the World Health Organization—specifically, the category M01A (nonsteroidal anti-inflammatory drugs), supplemented by standard pharmacological references (e.g., Goodman and Gilman’s Pharmacological Basis of Therapeutics, 14th ed.) [26].

For adverse events, the Standardized MedDRA Query (SMQ) for ulcer of the GI tract (SMQ code 20000106 in. MedDRA v26.0) was used. This SMQ encompasses 83 preferred terms related to GI ulcers. Thus, our analysis included the 21 ulcer-related preferred terms that had ≥1000 reports in the database.

### 4.3. Number of Reports on GI Tract Ulcers

The number of reported adverse events in cases in which the drug was used in the analysis was extracted. Table 1 shows the measurement results. The term adverse event was defined using the preferred terms shown in Table 3.

### 4.4. Relationship Between NSAIDs and GI Disorders

A 2 × 2 contingency table was created using Table B (Table 4); 0.5 was added to all cells as a Haldane correction. RORs and *p*-values were calculated using Fisher’s exact test for NSAIDs and adverse event terms related to GI disorders. Relative assessments were performed by detecting the signal represented by the ROR for individual drugs.

This method of imbalance analysis allowed us to identify cases with several adverse events reported for the drug. Based on the calculated values, a volcano plot was created with the ordinal logarithm of the inverse of the *p*-value (−log *p*) on the vertical axis and the natural logarithm of ROR, lnROR, on the horizontal axis [27,28,29,30,31] (Figure 2). A volcano plot was used to visually interpret the adverse events. Such scatter plots are often used to understand gene expression trends in microarray data analysis [32]. If the *p*-value was <1 × 10^−308^, it was not calculated using JMP Pro 18.0. Thus, −log *p* was set to 308 as an approximation. NSAIDs with statistically significant associations (*p* < 0.05) [27,28,29,30,31,33] with adverse events and >1000 reports were selected as the target drugs (31 types) for PCA. To investigate GI disorders by site, the preferred term terminology was classified into upper and lower-GI disorders.

The upper GI tract was defined as the region from the oral cavity to the duodenum and the lower GI tract as the area from the jejunum to the anus. We checked up to lower-level terms for any preferred terms that could not be classified, and those without a description of the site were defined as not classified.

### 4.5. PCA

In this study, the exhaustive analysis of all adverse events and medicines registered in FAERS required a method that could simultaneously capture numerous variables. Therefore, PCA was used as a method for evaluating data comprising multiple variables. PCA is a multivariate analysis method that is utilized for summarization. By compressing multidimensional data into a lower dimension, the overall association among variables can be validated, and a comprehensive index that captures the characteristics of the subjects can be obtained [13]. The principal component, a newly synthesized indicator, can be evaluated as a percentage of the total that the information it holds accounts for, with a contribution ratio that is calculated at the same time. The principal component values obtained can facilitate the creation of a scatter plot, referred to as a score plot, which allows for a visual understanding of the data characteristics. In this study, the correlation matrix was used to coordinate the principal components and obtain a standardized principal component score. It is also possible to calculate a value called the principal component loadings, which are the correlation coefficients between the principal components and the original variables. The loading vector of the loading plot using the principal component loadings can be used simultaneously with the score plot to understand the association between each data and variable.

### 4.6. Hierarchical Cluster Analysis

Clustering is a method for grouping rows with similar values based on multivariate data. HCA is a method for grouping similar data to create a classification [34]. In this study, hierarchical clustering using the ward method was performed with the loadings obtained via PCA and the target NSAIDs, which were set based on the dendrogram distance. The distance graph indexed the number of clusters, and the point at which the slope of the graph increased rapidly was adopted as the optimal number of clusters.

### 4.7. Statistical Analysis

All statistical analyses were performed using JMP Pro 18.0 (SAS Institute Inc., Cary, NC, USA), and a *p*-value < 0.05 indicated statistically significant differences.

## 5. Conclusions

Individual NSAIDs differ in terms of risk profiles for GI injury, including variations in the frequency, severity, and location of adverse events. In particular, COX-nonselective NSAIDs were found to be associated with more frequent but generally milder GI events. Meanwhile, COX-2-selective NSAIDs were associated with less frequent but more severe GI events. Nonetheless, future observational studies and randomized controlled trials should be performed to confirm these patterns, which can provide more granular data on NSAID-related GI effects. Moreover, future research should investigate dose-dependent effects, interactions with concomitant medications, and long-term clinical outcomes. Ultimately, the insights from this study may help inform the selection of NSAIDs and the implementation of appropriate prophylactic and monitoring strategies (e.g., by considering not only the incidence of GI injury but also the anatomical site and severity of the damage) to reduce the risk of GI injury in clinical practice.

## Figures and Tables

**Table 1:** Number of reports of NSAIDs for analysis.

Name of Drug	Number of Reports	Name of Drug	Number of Reports	Name of Drug	Number of Reports
Acetylsalicylic acid	766,502	Ketoprofen	15,902	Nimesulide	4893
Ibuprofen	263,957	Etodolac	11,815	Sulindac	3574
Rofecoxib	247,746	Valdecoxib	10,990	Flurbiprofen	2351
Naproxen	199,896	Etoricoxib	10,817	Epirizole	2232
Celecoxib	182,408	Salicylic acid	8627	Dexketoprofen	2068
Diclofenac	120,896	Nabumetone	8286	Benzydamine	2054
Meloxicam	60,806	Parecoxib	7113	Aceclofenac	1950
Metamizole	48,815	Mefenamic acid	7061	Oxaprozin	1615
Loxoprofen	46,514	Ketorolac	6315	Lornoxicam	1507
Piroxicam	32,406	Salicylamide	5590	Salsalate	1138
Indometacin	16,040				

**Table 2:** Sex and age of patients with gastrointestinal ulcers.

Sex	Patients with Gastrointestinal Ulcers	Patients Without Gastrointestinal Ulcers	*p*-Value (Fisher’s Exact Test)	ROR	95% CI
Male	61,978	19,077,011	<0.001	0.947	0.937–0.957
Female	89,080	28,957,601			
Unknown	13,980	3,571,783			
Age (years)	Gastrointestinal ulcers	Non-gastrointestinal ulcers	*p*-value (Fisher’s exact test)	ROR	95% CI
≥70	45,035	11,271,212	<0.001	0.774	0.766–0.784
<70	84,604	27,334,589			
Unknown	165,038	13,000,594			

CI: confidence interval.

**Table 3:** Number of reports of gastrointestinal ulcers.

Preferred Term	Number of Reports	Preferred Term	Number of Reports	Preferred Term	Number of Reports
Gastric ulcer	26,627	Hemorrhagic ulcer	5915	Gastrointestinal ulcer	2184
Duodenal ulcer perforation	23,916	Hemorrhagic duodenal ulcer	5216	Perforation of gastric ulcer	1601
Colitis ulcerative	19,013	Esophageal ulcer	4839	Rectal ulcer	1542
Ulcer	16,492	Peptic ulcer	4701	Erosive duodenitis	1289
Duodenal ulcer	10,697	Large intestinal ulcer	2624	Hemorrhagic gastrointestinal ulcer	1230
Gastric ulcer hemorrhage	9533	Erosive esophagitis	2523	Intestinal ulcer	1210
Gastritis erosive	7694	Hemorrhagic gastritis	2443	Ulcerative esophagitis	1134

**Table 4:** Two-by-two contingency table used to calculate the reporting odds ratio (ROR) and its corresponding *p*-value for NSAID-related gastrointestinal ulcer adverse events (rows: ulcer present/absent; columns: suspected drug used/not used).

	Gastrointestinal Ulcers	Non-gastrointestinal Ulcers
Suspected drug	a	b
Non-suspected drug	c	d

ROR, reporting odds ratio = (a/b)/(c/d).

## Data Availability

The original contributions presented in this study are included in the article/Supplementary Materials. Further inquiries can be directed to the corresponding author.

## Supplementary Materials

The following supporting information can be downloaded at https://www.mdpi.com/article/10.3390/ph18081204/s1: Table S1: Principal component loadings of all variables; Table S1 presents the principal component loadings for each variable, as determined by principal component analysis (PCA).

## Abbreviations

The following abbreviations are used in this manuscript:

HCA	Hierarchical cluster analysis
PCA	Principal component analysis
ROR	Reporting odds ratio
SMQ	Standardized MedDRA query
GI	Gastrointestinal
NSAIDs	Nonsteroidal anti-inflammatory drugs
COX	Cyclooxygenase
REAC	Reported adverse events by preferred term
DEMO	Demographic characteristics of the patients such as age, sex, and weight
DRUG	Drug information including name and administration route
OUTC	Outcomes
RPSR	Report sources
INDI	Indication
THER	Therapy details
CI	Confidence interval
PC	Principal component
GERD	Gastroesophageal reflux disease
