# Supplementary material for: Comprehensive Analysis of Gastrointestinal Injury Induced by Nonsteroidal Anti-Inflammatory Drugs Using Data from FDA Adverse Event Reporting System Database
Source: Pharmaceuticals (Basel). 2025 Aug 14;18(8):1204. doi: 10.3390/ph18081204 (PMC12388942; doi:10.3390/ph18081204)
Supplement: Supplementary file 1 [file pharmaceuticals-18-01204-s001.zip › pharmaceuticals-3782294-supplementary.pdf]

Table S1. presents the principal component loadings for each variable, as determined by principal component analysis (PCA.

Name of the drug	First principal component	Second principal component	Third principal component
ACECLOFENAC	0.56188	-0.32583	-0.5078
ACETYLSALICYLIC ACID	0.88238	0.11605	0.29387
BENZYDAMINE	0.66078	-0.44318	-0.28843
CELECOXIB	-0.13928	0.46812	0.14502
DEXKETOPROFEN	0.74683	-0.16811	-0.40962
DICLOFENAC	0.64751	0.43464	-0.35835
EPIRIZOLE	0.84067	-0.07745	-0.34626
ETODOLAC	0.55037	0.45232	-0.14275
ETORICOXIB	0.58555	0.27565	-0.35459
FLURBIPROFEN	0.73733	-0.15686	-0.23644
IBUPROFEN	0.66256	0.28328	0.31424
INDOMETACIN	0.68675	0.18731	0.09919
KETOPROFEN	0.64076	0.06159	-0.15314
KETOROLAC	0.7373	0.00694	-0.14874
LORNOXICAM	0.84659	0.13746	-0.10786
LOXOPROFEN	0.37635	0.55766	-0.15029
MEFENAMIC ACID	0.63573	0.29375	-0.36053
MELOXICAM	0.58374	0.61011	0.25184
METAMIZOLE	0.63377	0.21343	-0.03862
NABUMETONE	0.46872	-0.65888	0.20683
NAPROXEN	0.57758	0.44471	0.54551
NIMESULIDE	0.74153	-0.06667	0.09143
OXAPROZIN	0.50524	-0.59248	0.01399
PARECOXIB	0.66953	-0.1327	0.32866
PIROXICAM	0.49904	0.26299	0.2287
ROFECOXIB	0.26228	0.06972	0.73209
SALICYLAMIDE	0.56347	-0.19203	0.42744
SALICYLIC ACID	0.64448	-0.5329	0.30112
SALSALATE	0.75276	-0.38082	0.00106
SULINDAC	0.71407	-0.03442	0.02744
VALDECOXIB	0.55845	-0.37441	0.43692
